# Late-Onset Psoriatic Arthritis: Are There Any Distinct Characteristics? A Retrospective Cohort Data Analysis

**DOI:** 10.3390/life13030792

**Published:** 2023-03-15

**Authors:** Chrysoula G. Gialouri, Gerasimos Evangelatos, Alexios Iliopoulos, Maria G. Tektonidou, Petros P. Sfikakis, George E. Fragoulis, Elena Nikiphorou

**Affiliations:** 1 Joint Academic Rheumatology Program, Clinical Immunology-Rheumatology Unit, 2nd Department of Medicine and Laboratory, Medical School, National and Kapodistrian University of Athens, General Hospital of Athens “Hippokration”, 115 27 Athens, Greece; chr_gialou@yahoo.com; 2 Joint Academic Rheumatology Program, Rheumatology Unit, First Department of Propaedeutic Internal Medicine, Medical School, National and Kapodistrian University of Athens, “Laiko” General Hospital, 115 27 Athens, Greece; 3 Rheumatology Unit, 417 NIMTS Hospital, 115 21 Athens, Greece; 4 Institute of Infection, Immunity and Inflammation, University of Glasgow, Glasgow G12 8QQ, UK; 5 Centre for Rheumatic Diseases, School of Immunology and Microbial Sciences, King’s College Hospital, London SE1 9RT, UK; 6 Rheumatology Department, King’s College Hospital, London SE5 9RS, UK

**Keywords:** psoriatic arthritis, early-onset, late-onset, characteristics

## Abstract

As life expectancy increases, psoriatic arthritis (PsA) in older individuals becomes more prevalent. We explored whether late-onset versus earlier-onset PsA patients display different clinical features at diagnosis and/or during the disease course, as well as different treatment approaches and comorbidity profiles. We retrospectively collected data from consecutive PsA patients attending two rheumatology centers (December 2017–December 2022). Late-onset PsA patients (diagnosis-age: ≥60 years) were compared to those diagnosed before 60 years old. Univariate analyses and logistic regression were performed to examine for factors associated with late-onset PsA. For sensitivity analyses, the cohort’s mean diagnosis age was used as the cut-off value. Overall, 281 PsA patients were included (mean ± SD diagnosis-age: 46.0 ± 13.3 years). Of them, 14.2% (N = 40) had late-onset PsA. At diagnosis, after controlling for confounders, no demographic and clinical differences were identified. During the disease course, the late-onset group exhibited 65% fewer odds of manifesting enthesitis (adjusted Odds-ratio—adOR 0.35; 95% confidence interval 0.13–0.97), but higher frequency of dyslipidemia (adOR 3.01; 1.30–6.95) and of major adverse cardiovascular events (adOR 4.30; 1.42–12.98) compared to earlier-onset PsA group. No differences were found in the treatment approaches. In sensitivity analyses, PsA patients diagnosed after 46 (vs. ≤46) years old had an increased frequency of hypertension (adOR 3.18; 1.70–5.94) and dyslipidemia (adOR 2.17; 1.25–3.74). The present study underpins that late-onset PsA is not uncommon, while the age at PsA onset may affect the longitudinal clinical expression of the disease. Patients with late-onset PsA were less likely to manifest enthesitis but displayed increased cardiovascular risk.

## 1. Introduction

Psoriatic arthritis (PsA) is a chronic, inflammatory arthritis which is classified under the umbrella of spondyloarthritides (SpA). PsA is a highly heterogeneous disease, presenting with a variety of clinical manifestations. Apart from the skin, the peripheral joints and/or the spine, the entheses and other extra-musculoskeletal organs such as the eyes and bowel are commonly involved in PsA. In addition, patients with PsA exhibit higher cardiovascular morbidity and mortality compared to the general population. The pathogenesis of cardiovascular disease in PsA seems to be multifactorial, depending on several interrelated variables. These include traditional cardiovascular risk factors, both modifiable (e.g., hypertension, dyslipidemia, diabetes mellitus, and obesity, which are common comorbidities in PsA) and non-modifiable (e.g., gender, age, and genetic predisposition), as well as the effects of chronic underlying inflammation [1,2]. Furthermore, mental-health disorders (e.g., depression and anxiety) are often encountered in patients living with PsA and impose a significant burden over the disease course [3,4]. Considering all the above, many investigators have adopted the term “psoriatic disease” to reflect on the complexity of this clinical entity.

The prevalence of PsA varies by geographic region between 0.1–1% in the general population and 10–30% in patients with psoriasis [5,6], with a median time interval between the two diagnoses of about one decade [7]. Notably, PsA typically develops in patients with a preceding diagnosis of psoriasis, whereas the onset of arthritis occurs before the diagnosis of psoriasis only in about 15% of PsA cases [8]. The distribution of PsA is almost equal in males and females [9], while the incidence of PsA rises with aging, reaching a peak just before the age of 60 years and thereafter sharply declines [10].

Given the advancing aging of the population, the diagnosis of PsA in older individuals is becoming more prevalent in daily clinical practice [11,12]. In parallel, the management of PsA in this subgroup seems to be more complicated by the multimorbidity and the polypharmacy burden that comes along with this, in addition to the altered pharmacokinetics and/or pharmacodynamics [13,14,15]. Of note, it has been suggested that in psoriasis and in PsA, comorbidities might accumulate earlier than expected, introducing so-called “premature” aging [16,17,18]. Furthermore, the increased age-related risk of adverse events may influence therapeutic decisions, explaining the observed hesitancy for prescription of biologic agents in older PsA patients [19,20,21], although the available data from studies on patients with psoriasis or rheumatoid arthritis do not support any effect of the age on treatment efficacy [22,23].

In the context of psoriasis, the age of disease onset represents a well-defined covariate used to classify patients into two subpopulations, with distinct clinical and immunogenic patterns [24]. Accordingly, in PsA, there are limited data supporting that higher age of disease onset is associated with different genetic, histopathological, laboratory, and clinical traits, as well as with worse disease outcomes (e.g., more bone erosions) [16,25,26]. In addition, some investigators have estimated, through retrospective and cross-sectional studies with small sample sizes, that within the PsA populations there are differences in the prevalence of cardiovascular risk factors (e.g., of hypertension), reporting higher rates in patients with late-onset PsA than those with earlier disease onset [19,27].

In this retrospective cohort, we aimed to explore whether patients with late-onset PsA differ from those with earlier disease-onset, displaying distinct features at the time of PsA diagnosis and/or during the disease course, as well as different comorbidity profiles and treatment approaches.

## 2. Methods

### 2.1. Participants and Data Collection

Data were retrospectively collected from medical charts of consecutive patients with PsA diagnosis who attended the outpatient rheumatology clinic of two tertiary hospitals (“Laiko” general hospital, Athens and 417 “NIMTS” Army Shared Fund Hospital, Athens), from December 2017 up to December 2022. All patients included in this study fulfilled the CASPAR classification criteria [28]. Patients who had at least one follow-up visit were included; no other exclusion criteria were applied for this study.

The following data were recorded. (1) Demographic characteristics: gender, age at PsA diagnosis, body mass index (BMI), status (positive/negative) for HLA-B27, family history (i.e., first- and second-degree relatives) of psoriasis or SpA, and disease duration (time-interval between diagnosis and last follow-up visit). (2) PsA-related clinical characteristics present at the time of diagnosis and/or during the disease course (i.e., up to the last follow-up visit): axial disease (sacroiliitis and/or involvement of the cervical, thoracic, or lumbar spine; radiologically confirmed by X-ray or magnetic resonance imaging; and relevant clinical symptomatology), peripheral arthritis, the 68 tender joint count (TJC) and the 66 swollen joint count (SJC), the presence of active skin psoriasis, nail psoriasis, enthesitis, dactylitis, uveitis (confirmed by ophthalmologist), and inflammatory bowel disease (confirmed by colonoscopy). (3) Ever-present comorbidities (i.e., up to the last follow-up visit): hypertension (defined as systolic blood pressure ≥140 mmHg and/or diastolic blood pressure ≥90 mmHg in two measurements and/or prescription of antihypertensive medication), dyslipidemia (defined as total cholesterol >200 mg/dL and/or triglycerides >150 mg/dL and/or prescription of lipid-lowering therapy), diabetes mellitus (defined as fasting blood sugar ≥126 mg/dL and/or prescription of anti-diabetic medication), obesity (BMI ≥30 kg/m^2^), major adverse cardiovascular events (MACE, defined as a history of myocardial infarction, angina, and/or stroke), and depression (as indicated by the use of antidepressants prescribed by a psychiatrist). (4) PsA-related treatment history, recorded as the ever use of steroids (yes/no) and the total number of conventional-synthetic (cs) and biological (b) disease modifying antirheumatic drugs (DMARD) received from the time of PsA diagnosis. Of note, apremilast was included (for this study) in the latter group. Furthermore, in this cohort, no JAK inhibitors had been prescribed up to the last recorded visit.

### 2.2. Study Design and Statistical Analyses

For the main analyses, patients were categorized into two groups: those with late-onset PsA (i.e., age at diagnosis: ≥60 years) and those with earlier-onset PsA (i.e., patients diagnosed before the age of 60 years) as in previous PsA studies [26]. In addition, sensitivity analyses were performed, using the mean age at diagnosis of the overall cohort (Supplementary Figure S1) to dichotomize our sample. Patients with an age at PsA diagnosis of >46 years were compared to patients who were diagnosed at an age of ≤46 years.

Continuous variables are presented as mean ± standard deviation (SD) or median (interquartile range, IQR) if distributed normally or not, respectively, and categorical variables as absolute frequency (n) and percentage (%). Comparisons between the two PsA groups were made using the Student’s *t*-test or Mann–Whitney U test for the continuous variables (if distributed normally or not, respectively), and the chi-square test for the categorical variables.

For both main and sensitivity analyses, simple and multiple logistic regression were conducted to investigate for factors associated with late-onset PsA (dependent variable). The multivariable model was constructed including the gender, the disease duration and the variables which displayed significant differences in the univariable analyses. Firstly, a backward stepwise selection model was applied so as to find a reduced model which could best explain the data. Thereafter, we reran the multiple regression, including the same independent variables, using the enter method. In the case of the same results, the enter model was decided to be reported.

Statistical significance was considered for *p*-values less than 0.05. GraphPad Prism 9 and STATA 13.0 software were used.

### 2.3. Ethical Approval

This study was approved by the Institutional Review Board of “Laiko” (scientific council; number 780-21) and “NIMTS” hospital (scientific council; number 196-19). The study was conducted in accordance with the principles of the Declaration of Helsinki for human studies. Written informed consent was obtained from all participants.

## 3. Results

In total, 281 PsA patients were included, of whom most were females (58.0%) with a mean ± SD age at diagnosis of 46.0 ± 13.3 years (Supplementary Figure S1). Among them, 40 (14.2%) had late-onset PsA (52.5% males, mean ± SD age at diagnosis: 66.7 ± 5.3 years, Supplementary Figure S2) versus 241 (85.8%) patients who had been diagnosed before the age of 60 years (40.2% males, mean ± SD age at diagnosis: 42.5 ± 10.8 years, Supplementary Figure S2). Characteristics of this cohort groups are presented in detail in Table 1.

In the univariable analysis, no significant differences were identified in the comparison of gender, family history of psoriasis or spondyloarthritis, BMI, or smoking habits between the late- and earlier- onset PsA group. The disease duration was found to be shorter in the late- than the earlier-onset PsA group (median [IQR]: 21 [1.5–93.5] vs. 54 [18–142] months, *p* = 0.002).

At the time of diagnosis, patients with late-onset PsA had similar clinical manifestations to those diagnosed before the age of 60 years, in terms of axial disease, peripheral arthritis (including non-significant differences in the number of tender or swollen joints), active skin psoriasis lesions, enthesitis, dactylitis, and inflammatory bowel disease.

Over the disease course, patients with late-onset PsA manifested less frequently axial disease compared to those with earlier disease onset (22.5% vs. 39.0%, *p* = 0.045), whereas no significant differences were found for the other clinical features of PsA (Table 1). Notably, the percentage of patients who at least once exhibited enthesitis was lower in the late-onset PsA group. However, the difference was marginally non-significant (17.5% in the late-onset vs. 32.8% in the earlier-onset PsA group, *p* = 0.052). Furthermore, among the recorded comorbidities, hypertension, dyslipidemia, and MACE were significantly more frequent among patients with late-onset PsA (55% vs. 31%, *p* = 0.003; 75% vs. 48.1%, *p* = 0.002; 22.5% vs. 5.0%, *p* < 0.001, respectively). Finally, patients with late-onset PsA were found to have received an overall lower number of bDMARDs compared to those with an earlier disease onset (*p* = 0.035).

In multiple logistic regression (enter model, Table 2), having the gender, the disease duration, and the variables which displayed significant differences in univariate analyses as independent variables, patients with late- (vs. those with earlier-) PsA onset -who had a shorter disease duration- demonstrated 65% fewer odds of manifesting enthesitis during the disease course (adjusted Odds Ratio—adOR 0.35; 95% CI 0.13–0.97). On the other hand, patients with late-onset PsA had a 3-fold increased frequency of dyslipidemia (adOR 3.01; 95% CI 1.30–6.95, *p* = 0.010) and 4.3-fold increased frequency of MACE (adOR 4.30; 95% CI 1.42–12.98, *p* = 0.010) compared to patients who were diagnosed before 60 years old.

### Sensitivity Analyses

When we repeated the analyses using as the cut-off value the mean age at PsA diagnosis of the overall cohort (46 years), we did not find any differences in demographic characteristics and clinical features present at the time of diagnosis. Instead, in univariate comparisons, the frequency of uveitis during the disease course was found to be lower in the group of patients diagnosed after the age of 46 years, versus those with an age at diagnosis of ≤46 years (0.7% vs. 4.5%, *p* = 0.041). In addition, patients diagnosed after 46 years old were found to have a significantly increased frequency of hypertension (48.3% vs. 19.4%, *p* < 0.001), dyslipidemia (65.1% vs. 37.6%, *p* < 0.001), diabetes (24.8% vs. 13.4%, *p* = 0.004), and MACE (11% vs. 3.7%, *p* = 0.021). No difference was observed in the treatment approach between these groups. Details are presented in Supplementary Table S1.

However, after adjusting for confounders (i.e., gender, disease duration, and the above mentioned significantly associated factors from univariate analyses), it was found that patients who were diagnosed after 46 years old differed from those who were diagnosed at an age of ≤46 years in terms of a higher frequency of traditional cardiovascular risk factors only. In particular, the former group was about three times more likely to have hypertension (adOR 3.18; 95% CI 1.70–5.94; *p* < 0.001) and about two times more likely to have dyslipidemia (adOR 2.17; 95% CI 1.25–3.74; *p* = 0.005) (Supplementary Table S2).

## 4. Discussion

In older individuals, the clinical expression of PsA as well as the burden of comorbidities may differ from those with a younger age at disease onset, given the age-related pathophysiological alterations. Moreover, at the time of SpA onset, older patients may present with a mixture of features, some of which could resemble other rheumatic diseases with increased prevalence in older ages, challenging thus sometimes the diagnosis [29]. With this retrospective study, we aimed to add more evidence pertaining to whether patients with late-onset PsA differ from those with earlier PsA onset, at the time of diagnosis and/or during the disease course. We showed that patients with late-onset PsA had similar demographic and clinical characteristics at the time of diagnosis to those patients diagnosed before 60 years old, but were significantly less likely to manifest enthesitis during the disease course. In addition, patients with late-onset PsA displayed an increased cardiovascular risk, having more frequently dyslipidemia and MACE. Importantly, even patients diagnosed at a younger age (>46 years old vs. those diagnosed ≤46 years old) displayed an independent increased frequency of cardiovascular risk factors.

Previous studies on PsA patients investigating the influence of the age at the time of diagnosis on disease-related features are scarce. In addition, there is no consensus thus far on whether the age at disease onset should be analyzed as a continuous or binary variable, while in the latter case, different cut-off values have been used and so direct comparisons are not feasible [19,26,27,30,31,32]. In our main analyses, we included those patients who were diagnosed with PsA at the age of 60 years or older in the late onset group, in line with other studies conducted in the field. Besides, this is in agreement with the definition used by the United Nations for older persons [33]. In addition, recent findings from a population-based German study support that most of the new PsA cases occur between the age of 50 and 59 years, whereas the risk of developing PsA after 60 years becomes rapidly lower [10]. Notably, given that older persons are also referred to as those aged over 65 years old, we repeated the same analyses using this cut-off, but we found the same associations.

In our cohort, 14.2% of patients were diagnosed after the age of 60 years, which reflects the need to better characterize this PsA subgroup. In the main analyses, we found that patients with late-onset PsA had similar demographic characteristics as well similar clinical features at the time of diagnosis to those patients with earlier-onset PsA. Punzi et al. were the first to prospectively study a small (N = 66) cohort of PsA patients using the 60 years as the age threshold. In this study, the investigators suggested that patients with late-onset PsA had a more severe disease onset, with elevated inflammation markers in the serum and synovial fluid, more tender and/or swollen joints, and more bone erosions after two years of observation. On the other hand, in agreement with our findings, no differences were identified at the time of disease onset in terms of axial involvement (although the detection of sacroiliac joints involvement was made through bone scintigraphy in that study) and dactylitis presence. However, one has to note that in this study, a limited number of patients were included and only some of the clinical features of PsA were examined. Furthermore, a multivariable analysis that would correct for possible confounders was not applied [26].

In our dataset, we also estimated, after adjustment for confounders, that patients with late-onset PsA manifested less frequently enthesitis during the disease course, whereas no differences were observed in other ever-present clinical manifestations. In addition, patients with late-onset PsA showed an increased frequency of dyslipidemia and MACE compared to patients with earlier-onset PsA. On the other hand, no differences were identified in the treatment approach, as assessed in this study by the ever use of steroids and by the total number of ever received cs- or b-DMARDs. In agreement with our findings, a previous retrospective study of 180 PsA patients, with cut-off age for symptoms initiation at 65 years, showed that patients with late-onset PsA had a significantly higher rate of hypertension, diabetes mellitus, and coronary heart disease compared to those with early-onset PsA. Similarly to our findings, the analyses applied in this dataset showed that the late-onset group was also less likely to display enthesitis, but this group also manifested less frequently in dactylitis and nail psoriasis as well as in a greater skin psoriasis score. However, correction for possible confounders was not performed [19]. Furthermore, Queiro and colleagues conducted a cross-sectional study more recently that was based on a large sample of PsA patients (N = 227). This study also demonstrated (again only through univariate analyses) that patients with disease onset after the age of 65 years had a shorter disease duration and higher frequency of traditional cardiovascular risk factors (namely hypertension, dyslipidemia, and diabetes mellitus) compared to PsA groups diagnosed at a younger age. Moreover, patients with late-onset PsA were found to have a significantly lower use of bDMARDs, which is in concordance with our findings. However, in contrast to us, authors did not correct for cofounders and so the interpretation of this finding cannot be robust. Finally, in line with our results, no differences were found in the articular pattern (axial and/or peripheral), nor in the family history of psoriasis or PsA [27].

We also performed sensitivity analyses (Supplementary Tables S1 and S2), using the mean age at PsA diagnosis as the cut-off value of the overall cohort to define the late-onset group (>46 years old at diagnosis). We found that patients who were diagnosed after the age of 46 years had an independent increased cardiovascular risk, with a higher frequency of hypertension and dyslipidemia. On the other hand, these groups did not exhibit differences in the clinical expression of the disease at diagnosis a well as during the disease course, nor in the treatment options. This approach for defining the age threshold to compare early- and late-onset patients has not been used in previous PsA studies, but only in one retrospective study of patients with SpA [34].

The interpretation of the observed increased cardiovascular risk in the late-onset versus earlier-onset PsA group is complicated. One could speculate that there is an interplay between the higher age at PsA diagnosis and the cardiovascular burden. Given the underlying inflammatory nature of PsA upon its onset, PsA could promote or exacerbate the pre-existing age-related cardiovascular risk. On the other hand, it has been proposed that pro-inflammatory conditions such as adiposity, smoking, microbiome dysbiosis, immunosenescence, and “inflammageing”, as well as comorbidity-related drivers that may lead to a break in tolerance and to the corollary initiation of autoimmune inflammatory diseases later in life [12]. However, a lot of research is still required to fuel these theories.

We acknowledge that the present study has some limitations. Firstly, we had missing values which mainly pertained to the clinical features of patients at the time of diagnosis, given the retrospective nature of the study. Hence, the potential differences in disease presentation are still open to be explored. In addition, data for the exact date of comorbidities diagnosis were not available. The second limitation is consistent with previous studies and, as expected, is that the late-onset group had, significantly shorter disease duration and so other potential differences in clinical features during the disease course cannot be excluded. Thirdly, it is impossible to account for any residual confounding despite the multiple variables tested in the models. Finally, data for the date of psoriasis onset and for psoriasis duration up to the onset of joint disease are not available [35]. However, when we compared the frequency of patients in which psoriasis preceded the diagnosis of PsA, we did not observe a significant difference (65.9% in patients diagnosed before 60 years old and 62.5% in patients with late-onset PsA, *p* = 0.668).

Our study also has important strengths. First of all, this is the largest PsA sample used to compare late- and earlier-onset disease so far. In addition, we have included data both from the time of diagnosis and from the disease course, so as to capture differences at presentation as well as in the longitudinal clinical expression of the disease. Importantly, given the lack of a uniformly accepted definition of late-onset disease in the context of PsA, we also performed sensitivity analyses testing various values as the optimal cut-off for distinguishing the sub-group of late-onset PsA.

## 5. Conclusions

To conclude, in our cohort the late-onset PsA was not uncommon, while the age at PsA onset appeared to be another covariate which may affect the longitudinal clinical expression of the disease and thus needs to be considered in routine clinical practice. We found that patients with late-onset PsA had less frequent enthesitis during the disease course, but showed an increased cardiovascular risk compared to patients with earlier-onset PsA. Studies with longer follow-ups specifically designed to examine the characteristics of patients with late-onset PsA are required to further elucidate possible differences.

## Figures and Tables

**Table 1:** Characteristics of patients included in the study (N = 281). Comparison of patients with late-onset PsA (≥60 years) with those who were diagnosed before the age of 60 years.

	Earlier-Onset PsA (<60 Years) N = 241	Late-Onset PsA (≥60 Years) N = 40	*p*-Value
**Demographic characteristics**			
Gender, males, n (%)	97 (40.2)	21 (52.5)	0.146
Age at PsA diagnosis, mean ± SD	42.5 ± 10.8	66.7 ± 5.3	**<0.001**
BMI (kg/m^2^), mean ± SD	28.3 ± 5.7, N = 207 ^§^	28.5 ± 5.6, N = 32 ^§^	0.870
Family history of PsO, n (%)	66/213 (31.0) ^§^	10/37 (27.0) *	0.629
Family history of SpA, n (%)	10/207 (4.8) ^§^	3/36 (8.3) *	0.389
Smoking (ever/never), n (%)	105/228 (46.1 *)	17/36 (47.2) *	0.896
Disease duration (months), median (IQR)	54 (18–142)	21 (1.5–93.5)	**0.002**
**Clinical features at diagnosis**			
Axial disease, n (%)	46/217 (21.2) *	5/38 (13.2)	0.253
Peripheral arthritis, n (%)	193/209 (92.3) ^§^	34/35 (97.1)	0.302
Mono-/Oligo- arthritis, n (%)	72/183 (39.3)	16/33 (48.5)	0.325
68 TJC, median (IQR)	5.0 (3.0–7.0), N = 183 ^¥^	5.0 (3.0–8.5), N = 33 ^§^	0.146
66 SJC, median (IQR)	1.0 (2.0–4.0), N = 181 ^¥^	2.0 (2.0–3.5), N = 33 ^§^	0.874
Skin psoriasis, n (%)	122/165 (73.9) ^¥^	20/28 (71.4) ^¥^	0.781
Enthesitis, n (%)	23/169 (13.6) ^¥^	4/27 (14.8) ^¥^	0.866
Dactylitis, n (%)	23/173 (13.3) ^¥^	1/29 (3.5) ^¥^	0.129
Inflammatory bowel disease, n (%)	1 (0.4)	1 (2.5)	0.146
**Ever present clinical features**			
Axial disease, n (%)	94 (39.0)	9 (22.5)	**0.045**
Peripheral arthritis, n (%)	233 (96.7)	39 (97.5)	0.785
Skin psoriasis, n (%)	232 (96.3)	37 (92.5)	0.209
Nail psoriasis, n (%)	101 (41.9)	17 (42.5)	0.961
Enthesitis, n (%)	79 (32.8)	7 (17.5)	0.052
Dactylitis, n (%)	58 (24.1)	5 (12.5)	0.104
Uveitis, n (%)	7 (2.9)	0	0.275
Inflammatory bowel disease, n (%)	10 (4.1)	2 (5.0)	0.805
**Comorbidities**			
Hypertension, n (%)	74 (30.7)	22 (55.0)	**0.003**
Dyslipidemia, n (%)	115 (47.7)	30 (75.0)	**0.002**
Diabetes, n (%)	41/237 (17.3) *	10 (25.0)	0.245
Obesity, n (%)	64/207 (30.9) ^§^	9/32 (28.1) ^§^	0.750
MACE, n (%)	12 (5.0)	9 (22.5)	**<0.001**
Depression, n (%)	59/236 (25.0)	11 (27.5)	0.670
**Treatments ever received**			
csDMARDs (total number), median (IQR)	1 (1–2)	1 (1–1)	0.229
bDMARDs (total number), median (IQR)	1 (0–2)	0 (0–1)	**0.035**
Steroids, n (%)	120/226 (53.1) *	25 (62.5)	0.371

* Missing data ≤ 10%. ^§^ Missing data 10–20%. ^¥^ Missing data > 20%. Axial disease was defined as having sacroiliitis and/or spondylitis which were radiologically confirmed (by X-ray or magnetic resonance imaging) in addition to the relevant clinical symptomatology. Inflammatory bowel disease was confirmed by colonoscopy. Uveitis was confirmed by an ophthalmologist. Oligoarthritis was defined as the involvement of up to four joints. BMI—body mass index, TJC—tender joint count, SJC—swollen joint count, MACE—major adverse cardiovascular event, csDMARDs—conventional synthetic disease-modifying antirheumatic drugs, bDMARDs—biological DMARDs, n—number, SD—standard deviation, IQR—interquartile range. Significant differences are presented in bold.

**Table 2:** Simple and multiple logistic regression to assess for factors associated with late-onset PsA. Late- versus earlier-onset PsA.

	Crude OR (95% CI)	*p*-Value	Adjusted OR (95% CI)	*p*-Value
Female gender	0.60 (0.31–1.19)	0.149	0.66 (0.30–1.42)	0.290
Disease duration (months)	0.99 (0.98–0.99)	**0.027**	0.99 (0.98–0.99)	**0.012**
Axial disease (ever)	0.45 (0.20–0.99)	**0.049**	0.51 (0.21–1.20)	0.126
Enthesitis (ever)	0.43 (0.18–1.02)	0.057	0.35 (0.13–0.97)	**0.044**
Hypertension	2.72 (1.37–5.38)	**0.004**	1.72 (0.79–3.73)	0.170
Dyslipidemia	3.23 (1.51–6.51)	**0.002**	3.01 (1.30–6.95)	**0.010**
MACE	5.49 (2.14–14.1)	**<0.001**	4.30 (1.42–12.98)	**0.010**
bDMARDs (total number)	0.72 (0.51–1.01)	0.060	0.81 (0.55–1.20)	0.307

OR—odds ratio, CI—confidence interval, MACE—major adverse cardiovascular event, bDMARDs—biological disease-modifying antirheumatic drug. Significant differences are presented in bold.

## Data Availability

Data for this study are presented in the manuscript, tables, and Supplementary Material. Additional data are available upon request to the corresponding author.

## Supplementary Materials

The following supporting information can be downloaded at: https://www.mdpi.com/article/10.3390/life13030792/s1, Figure S1. Histogram of the age at PsA diagnosis of the entire cohort. Figure S2. Histogram of the age at PsA diagnosis by study group (late-onset vs. earlier-onset PsA). Table S1. Characteristics of patients included in the study (N = 281). Comparison of patients diagnosed after the age of >46 years with those who were diagnosed at an age of ≤46 years. Table S2. Simple and multiple logistic regression to assess for factors associated with late-onset PsA. Patients diagnosed after the age of >46 years versus those diagnosed at an age of ≤46 years.
