# Supplementary material for: Late-Onset Psoriatic Arthritis: Are There Any Distinct Characteristics? A Retrospective Cohort Data Analysis
Source: Life (Basel). 2023 Mar 15;13(3):792. doi: 10.3390/life13030792 (PMC10058512; doi:10.3390/life13030792)
Supplement: Supplementary file 1 [file life-13-00792-s001.zip › life-2226890-supplementary.pdf]

Supplementary material

Figure S1 Histogram of the age at PsA diagnosis of the entire cohort.

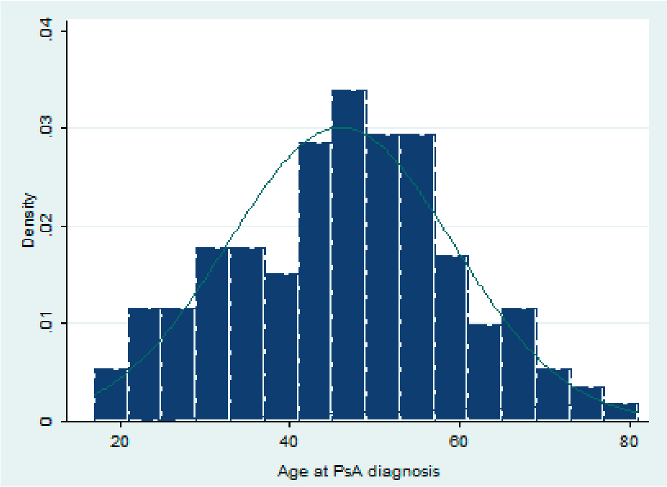

Figure S2 Histogram of the age at PsA diagnosis by study group (late-onset vs earlier-onset PsA).

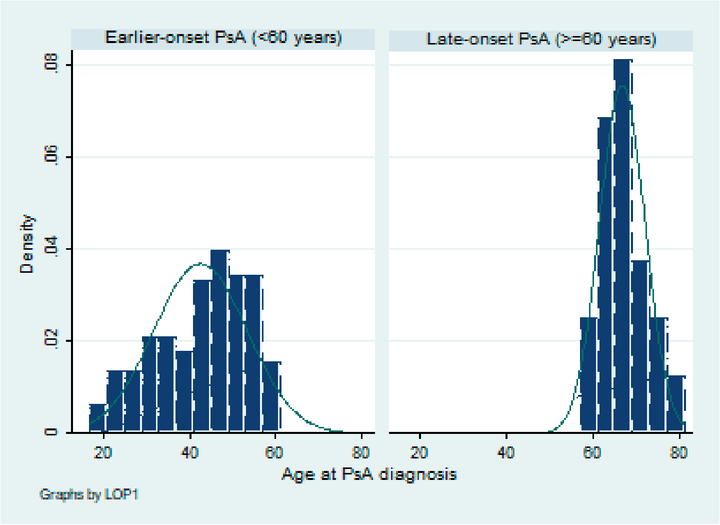

Table S1 Characteristics of patients included in the study (N=281). Comparison of patients diagnosed after the age of >46 years with those who were diagnosed at an age of ≤46 years.

	**Age at diagnosis ≤46 years N= 134**	**Age at diagnosis >46 years N= 147**	**p-value**
**Demographic characteristics**			
Gender, males, n (%)	60 (44.8)	58 (39.5)	0.367
Age at PsA diagnosis, mean ± SD	34.6 ± 8.0	56.1 ± 7.6	**<0.001**
BMI (kg/m^2), mean ± SD	27.8 ± 6.1	28.8 ± 5.1	0.187
Family history of PsO, n (%)	36/121 (29.8) *	40/129 (31.0) §	0.829
Family history of SpA, n (%)	4/116 (3.4) §	9/127 (7.1) §	0.208
Smoking (ever/never), n (%)	62/126 (49.2) *	60/138 (43.5) *	0.351
Disease duration (months), median (IQR)	74.0 (27.1-154.0)	33.5 (9.0-102.0)	**0.001**
**Clinical features at diagnosis**			
Axial disease, n (%)	26/115 (22.6) §	25/140 (17.9)*	0.345
Peripheral arthritis, n (%)	107/116 (92.2) §	120/128 (93.8) §	0.644
66/68 TJC, mean ± SD	5 (2-7)	5 (4-8)	0.196
66/68 SJC, median (IQR)	2 (1-4)	2 (2-4)	0.800
Skin psoriasis, n (%)	63/87 (72.4) ¥	79/106 (74.5) ¥	0.740
Enthesitis, n (%)	16/89 (18.0) ¥	11/107 (10.3) ¥	0.120
Dactylitis, n (%)	13/92 (14.1) ¥	11/110 (10.0) ¥	0.366
Inflammatory bowel disease, n (%)	1/132 (0.8)*	1/143 (0.7)*	0.955
**Ever present clinical features**			
Axial disease, n (%)	56 (41.8)	47 (32.0)	0.088
Peripheral arthritis, n (%)	129 (96.3)	143 (97.3)	0.631
Skin psoriasis, n (%)	127 (94.8)	142 (96.6)	0.633
Nail psoriasis, n (%)	49 (36.6)	69 (46.9)	0.070
Enthesitis, n (%)	46 (34.3)	40 (27.2)	0.196
Dactylitis, n (%)	33 (24.6)	30 (20.4)	0.397
Uveitis, n (%)	6 (4.5)	1 (0.7)	0.056
Inflammatory bowel disease, n (%)	4 (3.0)	8 (5.4)	0.309
**Comorbidities**			
Hypertension, n (%)	26 (19.4)	70/145 (48.3)*	**<0.001**
Dyslipidemia, n (%)	50/133 (37.6)*	95/146 (65.1)*	**<0.001**
Diabetes, n (%)	15/132 (13.4)*	36/145 (24.8)*	**0.004**
Obesity, n (%)	32/117 (27.4) §	41/122 (33.6) §	0.294
MACE, n (%)	5 (3.7)	16/145 (11.0)*	**0.021**
Depression, n (%)	32/131 (24.4)*	38/143 (26.6)*	0.684
**Treatments ever received**			
csDMARDs (total number), median (IQR)	1 (1-2)	1 (1-1)	0.146
bDMARDs (total number), median (IQR)	1 (0-2)	1 (0-2)	0.077
Steroids, n (%)	66/129 (51.2)*	79/136 (58.1)*	0.527

*Missing data ≤10%.
§Missing data 10-20%.
¥Missing data >20%.
Axial disease was defined as having sacroiliitis and/or spondylitis, which were radiologically confirmed in addition to relevant clinical symptomatology. Inflammatory bowel disease was confirmed by colonoscopy. BMI; body mass index, TJC; tender joint count, SJC; swollen joint count, MACE; major adverse cardiovascular event, csDMARDs; conventional synthetic disease modifying antirheumatic drugs, bDMARDs; biologic DMARDs, n; number, SD; standard deviation, IQR; interquartile range. Significant differences are presented in bold.

Table S2 Simple and multiple logistic regression to assess for factors associated with late-onset PsA. Patients diagnosed after the age of >46 years versus those diagnosed at an age of ≤46 years.

	**Crude OR (95% CI)**	**p-value**	**Adjusted OR (95% CI)**	**p-value**
Female sex	1.22 (0.76-1.96)	0.406	1.41 (0.82-2.42)	0.201
Disease duration (months)	0.997 (0.994-0.999)	**0.018**	0.995 (0.992-0.998)	**0.002**
Hypertension	3.84 (2.24-6.57)	**<0.001**	3.18 (1.71-5.91)	**<0.001**
Dyslipidemia	3.05 (1.87-4.98)	**<0.001**	2.26 (1.31-3.89)	**0.003**
Diabetes	2.76 (1.41-5.39)	**0.003**	1.52 (0.70-3.31)	0.282
MACE	3.17 (1.12-8.92)	**0.028**	1.76 (0.57-5.40)	0.319

OR; odds ratio, CI; confidence interval, MACE; major adverse cardiovascular event. Significant differences are presented in bold.
